# Quality of life scores differs between genotypic groups of patients with suspected hereditary hemochromatosis

**DOI:** 10.1186/s12881-017-0513-5

**Published:** 2018-01-05

**Authors:** Paula Fernanda Silva Fonseca, Rodolfo Delfini Cançado, Flavio Augusto Naoum, Carla Luana Dinardo, Guilherme Henrique Hencklain Fonseca, Sandra Fatima Menosi Gualandro, José Eduardo Krieger, Alexandre Costa Pereira, Pierre Brissot, Paulo Caleb Junior Lima Santos

**Affiliations:** 1 Laboratory of Genetics and Molecular Cardiology, Heart Institute (InCor), University of São Paulo Medical School, Av. Doutor Enéas de Carvalho Aguiar, 44-Cerqueira César, São Paulo, 05403 900 Brazil; 2 Hematology and Hemotherapy Section, Santa Casa Medical School, São Paulo, Brazil; 3 Academia de Ciência e Tecnologia, São José do Rio Preto, Brazil; 4 Fundação Pró-Sangue, Hemocentro de São Paulo, São Paulo, SP Brazil; 5 Universidade de São Paulo (USP), São Paulo, SP Brazil; 6 Hematology Service, Hospital das Clinicas, Medical School, University of São Paulo, São Paulo, Brazil; 7 Hematology and Hemotherapy Discipline, Hospital das Clinicas, Medical School, University of São Paulo, São Paulo, Brazil; 8 Liver Disease Unit, Pontchaillou University Hospital, University of Rennes, and National Reference Centre for Rare Iron Overload Diseases of Genetic Origin, Rennes, France; 9 Department of Pharmacology, Universidade Federal de Sao Paulo - UNIFESP, São Paulo, Brazil

**Keywords:** Hereditary hemochromatosis, Quality of life, Short form health survey, SF-36

## Abstract

**Background:**

Hereditary hemochromatosis (HH) encompasses a group of autosomal recessive disorders mainly characterized by enhanced intestinal absorption of iron and its accumulation in parenchymal organs. HH diagnosis is based on iron biochemical and magnetic resonance imaging (MRI) assessment, and genetic testing. Questionnaires, such as SF-36 (short form health survey), have been increasingly used to assess the impact of diseases on the patient’s quality of life (QL). In addition, different genotypes are identified as results of genetic tests in patients with suspected primary iron overload. In the present study, our aim was to evaluate whether domains of QL are different according to genotypic groups in patients suspected of HH.

**Methods:**

Seventy-nine patients with primary iron overload were included and two genotypic groups were formed (group 1: homozygous genotype for the *HFE* p.Cys282Tyr mutation; group 2: other genotypes).

**Results:**

Group 1 had higher means of plasma transferrin saturation (86 ± 19%) and serum ferritin (1669 ± 1209 ng/mL) compared to group 2 (71 ± 12%, 1252 ± 750 ng/mL, respectively; *p* = 0.001). Four domains were significantly different among groups 1 and 2: physical functioning (*p* = 0.03), bodily pain (*p* = 0.03), vitality (*p* = 0.02) and social functioning (*p* = 0.01).

**Conclusions:**

Our main finding was that patients with p.Cys282Tyr homozygosity had a worse QL scenario assessed by SF-36, compared with patients with iron overload without the same genotype. Being aware of this relationship between genotypes and QL might be helpful in the overall management of patients suspected of hereditary hemochromatosis.

**Electronic supplementary material:**

The online version of this article (doi: 10.1186/s12881-017-0513-5) contains supplementary material, which is available to authorized users.

## Background

Hereditary hemochromatosis (HH) is an autosomal recessive disorder mainly characterized by enhanced intestinal absorption of iron and its accumulation in parenchymal organs. The main symptoms of HH are fatigue, skin pigmentation, joint pains, impotence, liver signs, diabetes, and cardiac symptoms [1–4]. HH is classified in 5 types. Type 1 is related to *HFE* mutations, especially by homozygous genotype for the p.Cys282Tyr mutation. Other HH types are named non-*HFE* hemochromatosis: type 2 or juvenile hemochromatosis (JH), sub-divided into 2 forms, type 2A, related with mutations in the *HJV* gene, and type 2B, related with mutations in the *HAMP* gene; type 3 is related to mutations in the *TFR2* gene*;* and type 4 is related to mutations in the *SLC40A1* gene [3, 4].

The diagnosis is essentially based on iron assessment by laboratory tests, magnetic resonance imaging (MRI), and genetic testing. p.Cys282Tyr homozygosity in patients with iron overload is the most frequent finding. However, other genotypes, such as compound heterozygosity for the p.Cys282Tyr/p.His63Asp mutations, heterozygosity or a negative result for these two genetic variants, can also be observed in patients with suspected HH iron overload [5, 6].

Quality of life (QL) is a widely used concept in scientific research in the fields of economy, education, medicine, psychology and health sciences. The term QL was firstly cited in the medical literature in the 30s [7, 8]. According to the World Health Organization (WHO), QL is “the individual’s perception of their position in life, in the context of culture and value systems in which they live and in relation to their goals, expectations, standards and concerns” [9]. Generic or specific instruments can assess QL. Generic instruments evaluate the aspects related with QL of patients in global or generic forms, while the specific instruments assess QL in an area of interest, such as a specific disease, population, condition or situation [10–12].

The short form health survey (SF-36) is a generic and standardized QL questionnaire. The explored domains are derived from forty domains of the Medical Outcomes Study [13]. The SF-36 has several strengths, such as versatility and the small time of administration [13–15]. The questionnaire was translated and validated in the Portuguese language, and adapted culturally for the Brazilian population [14, 16]. Our hypothesis that QL would differ according to genotypes is based on the risk of iron overload among genotypes. These differences and interpretation of the genotypes were described by *European Molecular Genetics Quality Network (EMQN),* an organization that promote quality in genetic testing by establishing, harmonizing and disseminating best practice [2].

Our aim was to explore whether domains of QL, as evaluated by the SF-36, were different according to genotypic groups in patients with suspected HH.

## Methods

### Patients

The study protocol was approved by the Institutional Ethics Committee of Instituto do Coração - HC-FMUSP (4027/14/007) and written informed consent was obtained from all participants prior to entering the study, in compliance with the Helsinki Declaration. Patients were selected from hematology outpatient clinics (Ambulatório de Hematologia do Hospital das Clínicas - São Paulo, Ambulatório do Hemocentro da Santa Casa - São Paulo, and Instituto Naoum de Hematologia - São José do Rio Preto), Brazil.

The inclusion criteria were: age greater than or equal to 18 years, transferrin saturation (TS) ≥45% and serum ferritin (SF) ≥200 ng/mL for females or ≥300 ng/mL for males. The exclusion criteria were: patients with positive serology for hepatitis C or B, alcoholic liver disease, high alcoholic consumption (>20 g daily), hemolytic anemias, repeated blood transfusions, metabolic syndrome, or insulin resistance not resulting from HH. In this analyzed casuistic, we also excluded patients with juvenile hemochromatosis carrying *HAMP* and *HJV* mutations [17, 18].

### DNA extraction and genetic testing

Blood was drawn using the BD Vacutainers System® (Becton Dickinson, NJ, USA) for blood cell count and genetic analysis. Genomic DNA was isolated from peripheral blood leukocytes by a salting-out method. Coding sequences of the *HFE* exons 2 and 4 were amplified by polymerase chain reaction (PCR) using the previously described primer sequences [19]. PCR products were purified using ExoSAP-IT® reagent (GE Healthcare, NJ, USA) and were bidirectionally sequenced using the ABI Terminator Sequencing Kit according to the manufacturer’s instructions and an ABI 3500XL Sequencer® (Applied Biosystems, Foster City, CA, USA).

### SF-36 health survey

The questionnaire is based on 36 questions. It is distributed in eight domains representing the most frequently evaluated and influenced by diseases and/or treatments, consisting of physical functioning, role-physical, bodily pain, general health perception, vitality, social functioning, role-emotional and mental health. The values for each domain range from 0 to 100 points; and higher scores indicate a better health condition [13–15].

All participants of both groups answered the SF-36 questionnaire once, applied by an interviewer or self-administered plus reviewed by an interviewer. The questionnaire was applied after the knowledge of the genotype by the patient. For each question, fixed values were attributed and then converted in eight scores (raw scale) for the domains. Thus, eight values were obtained, one for each domain. The scoring of SF-36 was performed according to the instructions of the instrument authors [15].

### Statistical analysis

Categorical variables are presented as percentages, while continuous variables are presented as means ± standard deviations. Kolmogorov-Smirnov test was used for testing Normality. Chi-square or Fisher tests were performed for the comparative analysis of the categorical variables (such as gender, self-declared ethnicity, level of education, and consumption of alcoholic beverages) according to the genotypic group. Student’s t-test was used for comparing mean values of the SF-36 domains, TS and SF among groups 1 and 2. Mean values of the TS and SF were adjusted for age and gender. Mean values of the SF-36 domains were adjusted for age, gender, TS and SF. The level of significance was set at *p* ≤ 0.05. All statistical analyses were carried out using SPSS software (v. 16.0).

## Results

Seventy-nine patients with primary iron overload were included. Two genotypic groups were formed: group 1, patients with primary iron overload and homozygous genotype for the p.Cys282Tyr mutation (*n* = 29). And, group 2, patients with primary iron overload and other genotypes: compound heterozygosity for the p.Cys282Tyr/p.His63Asp mutation (*n* = 11), heterozygosity for the p.Cys282Tyr (*n* = 4), homozygosity (*n* = 12) or heterozygosity (*n* = 9) for the p.His63Asp, or absence of p.Cys282Tyr or p.His63Asp (*n* = 14).

Table 1 shows the general characteristics according to genotypic groups of the patients. Group 1 presented lower percentage of males (44.8%) and higher means of TS (86 ± 19%) and SF (1669 ± 1209 ng/mL) compared with group 2 (84.0%, 71 ± 12%, 1252 ± 750 ng/mL; 0.001, < 0.001, < 0.001, respectively). We did not observe significant difference in a comparison of frequency of the common diseases and of therapy phases among patient groups.

**Table 1:** General characteristics according to genotypic groups of the patients

	Group 1^a^, *n* = 29	Group 2^b^, *n* = 50	*p* value
Gender (male), %	44.8	84.0	0.001
Age (years), mean ± SD	45 ± 12	49 ± 12	0.14
Transferrin saturation (%), mean ± SD	86 ± 19	71 ± 12	< 0.001
Serum ferritin (ng/mL), mean ± SD	1669 ± 1209	1252 ± 750	< 0.001
Self-declared race/color, %			
White	82.7	82.0	
Intermediate	17.3	12.0	0.35
Others	0	6.0	
Level of education, %			
University	62.1	68.0	0.77
Others	37.9	32.0	
Consumption of alcoholic beverages, %			
Never	51.7	36.0	
Occasionally	44.8	54.0	0.30
Frequently	3.5	10.0	

^a^Group 1: patients with primary iron overload and homozygosity for the p.Cys282Tyr mutation

^b^Group 2: patients with primary iron overload and other genotypes: compound heterozygosity for the p.Cys282Tyr/p.His63Asp (*n* = 11), heterozygosity for the p.Cys282Tyr (*n* = 4), homozygosity (*n* = 12) or heterozygosity (*n* = 9) for the p.His63Asp, or absence of p.Cys282Tyr or and p.His63Asp (*n* = 14)

Table 2 shows the mean values of the SF-36 domains according to patient groups. Four domains were significantly different among groups 1 and 2: physical functioning (*p* = 0.03), bodily pain (p = 0.03), vitality (*p* = 0.02) and social functioning (*p* = 0.01). Group 1 had lower mean values for these four domains compared with group 2 (Table 2). In addition, Additional file 1: Table S1 shows the signs and symptoms reported by patients according to the genotypic groups. Group 1 reported greater frequency of signs and symptoms compared with group 2.

**Table 2:** Mean (±standard deviation) values of the SF-36 domains according to genotypic groups of the patients

SF-36 domains	Group 1, *n* = 29	Group 2, *n* = 50	*p* value
Physical functioning	78 ± 23	90 ± 14	0.03
Role-physical	75 ± 40	83 ± 31	0.36
Bodily pain	66 ± 25	78 ± 22	0.03
General health perception	62 ± 23	67 ± 14	0.41
Vitality	53 ± 26	67 ± 18	0.02
Social functioning	69 ± 35	85 ± 17	0.01
Role-emotional	77 ± 34	82 ± 31	0.53
Mental health	67 ± 24	74 ± 17	0.18

## Discussion

The most common, well-defined and prevalent form of HH is *HFE*-related HH, associated with homozygosity for the p.Cys282Tyr. However, there are other genotypes, which have lower penetrance for HH, that are relatively frequent in patients with iron overload [2, 20]. In this study, we defined group 1 as the one formed by patients carrying homozygous genotypes for the p.Cys282Tyr mutation and group 2 with patients carrying other genotypes. Group 1 had lower values for four domains (physical functioning, bodily pain, vitality and social functioning) compared with group 2. Therefore, patients with p.Cys282Tyr homozygosity had worse QL assessed by SF-36, compared with patients with iron overload without the same genotype. Both groups 1 and 2 of patients with iron overload require therapeutic management, but the knowledge of this relationship between identified genotype and QL might be an interesting information for improving the clinical care of the patient, including physical and social aspects.

Some interesting studies reported the SF-36 use with HH patients. Meiser et al. studied the difference among clinically affected and unaffected subjects in a hemochromatosis clinic in Australia. Thirty participants were categorized as being clinically affected, and sixty-six as clinically unaffected. For all domains of SF-36, clinically unaffected individuals had higher scores than affected individuals [21]. Van der Plas et al. investigated patients of the Dutch liver patient association divided into five groups: hemochromatosis, viral hepatitis, autoimmune hepatitis, cholestatic liver disease, and other liver diseases. They showed that patients with HH had significantly lower scores in the domain of bodily pain compared with patients with other etiological groups. In addition, HH patients had significantly lower scores in the physical component of all other etiological groups, except in relation to autoimmune hepatitis [22]. Graaff et al., using the Assessment of Quality of Life 4D instrument (AQOL-4D), assessed 270 patients with HH that completed a web-based survey in Australia. This questionnaire provides a global health state utility value on a scale from −0.04 to 1.00. The mean utility for all HH participants was 0.66, lower than the Australian population (0.81). In addition, they observed that symptomatic stages of HH were associated with lower utility than asymptomatic stages [23]. In contrast to the findings of the present study and to above-mentioned studies, Shaheen et al., recruiting 126 HH patients from outpatient clinics, demonstrated that subjects with HH reported a QL similar to both unaffected siblings and general population [24].

In the present study, we observed greater proportion of males in the group 2 compared with group 1. This may be related to the known relative protection for iron overload by menstruations and pregnancies added to low penetrance associated to genotypes other than non-p.Cys282Tyr homozygosity. A possible influence of the high percentage of women in the group 1 on the observed lower scores cannot be excluded [25]. However, we were able to identify significant findings performing an adjusted analysis for the covariates age and gender.

In addition, group 1 had higher TS and SF mean values compared with group 2. Nevertheless, the mean values of group 2 were also very high, compared with iron parameters in the general population. The observed differences could be explained by group classification based on the genotypes. The current interpretation of genotypes and their influence on iron overload has been detailed by the EMQN guidelines. p.Cys282Tyr homozygosity is compatible with *HFE*-related HH in the presence of documented evidence of iron overload; patients with Cys282Tyr/p.His63Asp compound heterozygosity may be at-risk of developing mild to moderate iron overload in association with comorbid factors (such as metabolic syndrome or chronic alcoholism); heterozygous p.Cys282Tyr; homozygous p.His63Asp, and heterozygous p.His63Asp carriers have no increased risk of developing *HFE*-related HH; are at no increased risk of developing *HFE*-related HH. Testing for the p.S65C variant is not recommended [2].

Our study has some limitations. First, MRI was not performed for organ iron overload quantification. However, the inclusion criteria that combined elevated SF and TS together with exclusion criteria ruling out the interference of the secondary causes, strongly favor the diagnosis of primary iron overload in the whole cohort. Second, we tested only the main mutations in the *HFE* gene, thus, we cannot exclude the presence of other mutations in the *HFE*, *HJV* (type 2A HH), *HAMP* (type 2B HH), *TFR2* (type 3 HH), and *SLC40A1* (type 4B) genes. However, there was no patient with JH profile in this casuistic. Finally, domain differences could not be related to numerical differences of the signs and symptoms.

## Conclusions

In conclusion, this study mainly shows that patients with p.Cys282Tyr homozygosity had worse QL scenario assessed by SF-36, compared with patients with primary iron overload that do not carry the same genotype. Being aware of this relationship between genotypes and QL might be helpful in the overall management of patients suspected of hereditary hemochromatosis.

## Additional file

Additional file 1: Table S1. Signs and symptoms reported by patients according to genotypic groups. (DOCX 14 kb)

## References

[1] Brissot P (2016). Optimizing the diagnosis and the treatment of iron overload diseases. Expert Rev Gastroenterol Hepatol.

[2] Porto G, Brissot P, Swinkels DW, Zoller H, Kamarainen O, Patton S (2016). EMQN best practice guidelines for the molecular genetic diagnosis of hereditary hemochromatosis (HH). Eur J Hum Genet.

[3] Santos PC, Dinardo CL, Cancado RD, Schettert IT, Krieger JE, Pereira AC (2012). Non-HFE hemochromatosis. Rev Bras Hematol Hemoter.

[4] Santos PC, Krieger JE, Pereira AC (2012). Molecular diagnostic and pathogenesis of hereditary hemochromatosis. Int J Mol Sci.

[5] Santos PC, Cancado RD, Pereira AC, Schettert IT, Soares RA, Pagliusi RA, et al. Hereditary hemochromatosis: mutations in genes involved in iron homeostasis in Brazilian patients. Blood Cells Mol Dis. 46(4):302–7. doi:S1079-9796(11)00060-X 10.1016/j.bcmd.2011.02.008 10.1016/j.bcmd.2011.02.008 21411349

[6] Santos PC, Pereira AC, Cancado RD, Schettert IT, Sobreira TJ, Oliveira PS (2010). HFE gene mutations in patients with primary iron overload: is there a significant improvement in molecular diagnosis yield with HFE sequencing?. Blood Cells Mol Dis.

[7] Rogerson RJ (1995). Environmental and health-related quality of life: conceptual and methodological similarities. Soc Sci Med.

[8] Seidl EMF, Zannon CM (2004). Qualidade de vida e saúde: aspectos conceituais e metodológicos. Cadernos de Saúde Pública.

[9] The World Health Organization Quality of Life assessment (WHOQOL) (1995). Position paper from the World Health Organization. Soc Sci Med.

[10] Campolina AG, Dini PS, Ciconelli RM (2011). Impacto da doença crônica na qualidade de vida de idosos da comunidade em São Paulo (SP, Brasil). Ciência Saúde Coletiva.

[11] Campos MO, Neto JFR (2008). Qualidade de vida: um instrumento para promoção de saúde. Revista Baiana de Saúde Pública.

[12] Guyatt GH, Feeny DH, Patrick DL (1993). Measuring health-related quality of life. Ann Intern Med.

[13] Ware JE (2000). SF-36 health survey update. Spine (Phila Pa 1976).

[14] Campolina AG, Ciconelli RM (2008). O SF-36 e o desenvolvimento de novas medidas de avaliação de qualidade de vida. Acta Reumatol Port.

[15] Ware JE, S K, Kosinski M, Gandek B. SF-36 health survey manual and interpretation guide. In: The health institute. Boston; 1993.

[16] Ciconelli RM, F M, Santos W, Meinão I, Quaresma MR (1999). Tradução para a língua portuguesa e validação do questionário genérico de avaliação de qualidade de vida SF-36 (Brasil SF-36). Rev Bras Reumatol.

[17] de Lima Santos PC, Pereira AC, Cancado RD, Schettert IT, Hirata RD, Hirata MH (2010). Hemojuvelin and hepcidin genes sequencing in Brazilian patients with primary iron overload. Genet Test Mol Biomarkers.

[18] Fonseca PF, Cancado RD, Uellendahl Lopes MM, Correia E, Lescano MA, Santos PC (2016). HAMP gene mutation associated with juvenile Hemochromatosis in Brazilian patients. Acta Haematol.

[19] Santos PC, Soares RA, Krieger JE, Guerra-Shinohara EM, Pereira AC. Genotyping of the hemochromatosis HFE p.H63D and p.C282Y mutations by high-resolution melting with the rotor-gene 6000(R) instrument. Clin Chem Lab Med. 49(10):1633–6. doi: 10.1515/CCLM.2011.654. 10.1515/CCLM.2011.654 21679129

[20] Cancado R, Melo MR, de Moraes Bastos R, Santos PC, Guerra-Shinohara EM, Chiattone C (2015). Deferasirox in patients with iron overload secondary to hereditary hemochromatosis: results of a 1-yr phase 2 study. Eur J Haematol.

[21] Meiser B, Dunn S, Dixon J, Powell LW (2005). Psychological adjustment and knowledge about hereditary hemochromatosis in a clinic-based sample: a prospective study. J Genet Couns.

[22] van der Plas SM, Hansen BE, de Boer JB, Stijnen T, Passchier J, de Man RA (2007). Generic and disease-specific health related quality of life of liver patients with various aetiologies: a survey. Qual Life Res.

[23] de Graaff B, Neil A, Sanderson K, Yee KC, Palmer AJ (2016). Quality of life utility values for hereditary haemochromatosis in Australia. Health Qual Life Outcomes.

[24] Shaheen NJ, Lawrence LB, Bacon BR, Barton JC, Barton NH, Galanko J (2003). Insurance, employment, and psychosocial consequences of a diagnosis of hereditary hemochromatosis in subjects without end organ damage. Am J Gastroenterol.

[25] Laguardia J, Campos MR, Travassos C, Najar AL, Anjos LA, Vasconcellos MM (2013). Brazilian normative data for the short form 36 questionnaire, version 2. Rev Bras Epidemiol.

