# Supplementary material for: Quality of life scores differs between genotypic groups of patients with suspected hereditary hemochromatosis
Source: BMC Med Genet. 2018 Jan 5;19:3. doi: 10.1186/s12881-017-0513-5 (PMC5755339; doi:10.1186/s12881-017-0513-5)
Supplement: Additional file 1: Table S1. — Signs and symptoms reported by patients according to genotypic groups. (DOCX 14 kb) [file 12881_2017_513_MOESM1_ESM.docx]

**Supplementary table**

**Supplementary table 1** **–** Signs and symptoms reported by patients according to genotypic groups.

| **Signs and symptoms*** | **Group 1, n= 29** | **Group 2, n= 50** | **p value** |
| --- | --- | --- | --- |
| Joint pain, n (%) | 9 (31) | 7 (14) | 0.07 |
| Weakness, n (%) | 8 (28) | 12 (24) | 0.72 |
| Brittle nails and hair, n (%) | 8 (28) | 8 (16) | 0.22 |
| Somnolence, n (%) | 8 (28) | 8 (16) | 0.22 |
| Irritability, n (%) | 7 (24) | 7 (14) | 0.26 |
| Dizziness, n (%) | 6 (21) | 4 (8) | 0.16 |
| Headache, n (%) | 6 (21) | 3 (6) | 0.07 |
| Abdominal pain, n (%) | 5 (17) | 4 (8) | 0.28 |
| Weight loss, n (%) | 4 (14) | 5 (10) | 0.72 |
| Sweating, n (%) | 4 (14) | 3 (6) | 0.41 |
| Fatigue, n (%) | 3 (10) | 5 (10) | 1.00 |
| Palpitation, n (%) | 3 (10) | 3 (6) | 0.66 |
| Constipation and diarrhea, n (%) | 3 (10) | 2 (4) | 0.35 |
| Skin hyperpigmentation, n (%) | 2 (7) | 4 (8) | 1.00 |
| Sexual problems, n (%) | 2 (7) | 1 (2) | 0.55 |

* The most frequent signs and symptoms were showed.
